# The Effect of Perceived Parent–Child Facial Resemblance on Parents’ Trait Anxiety: The Moderating Effect of Parents’ Gender

**DOI:** 10.3389/fpsyg.2016.00658

**Published:** 2016-05-04

**Authors:** Quanlei Yu, Qiuying Zhang, Jianwen Chen, Shenghua Jin, Yuanyuan Qiao, Weiting Cai

**Affiliations:** ^1^Beijing Key Lab of Applied Experimental Psychology, School of Psychology, Beijing Normal UniversityBeijing, China; ^2^Department of Teaching and Learning School of Education and Human Development, University of Miami, MiamiFL, USA; ^3^Department of Developmental and Educational Psychology, Graduate School of Education, Huazhong University of Science and TechnologyWuhan, China; ^4^Department of Psychology, School of Teacher Education, Shaanxi Normal UniversityLinfen, China; ^5^Kindergarten of Beijing Military RegionBeijing, China

**Keywords:** Parent–child facial resemblance, trait anxiety, parent gender, evolution psychology, uncertainty of paternity

## Abstract

Father–child facial resemblance is an important cue for men to evaluate paternity. Previous studies found that fathers’ perceptions of low facial resemblance with offspring lead to low confidence of paternity. Fathers’ uncertainty of paternity could cause psychological stress and anxiety, which, after a long time, may further turn into trait anxiety. Conversely, females can ensure a biological connection with offspring because of internal fertilization. The purpose of this study was thus to examine the role of parents’ gender in the effect of parents’ perceived facial resemblance with child on their trait anxiety. In this study, 151 parents (father or mother) from one-child families reported their facial resemblance with child and their trait anxiety. Results showed that (i) males tended to perceive higher facial similarity with child than did females and (ii) males’ perceived facial resemblance with child significantly predicted trait anxiety, whereas females’ perceived facial resemblance did not. These findings suggested that the uncertainty of paternity contributed to the trait anxiety of fathers, but not mothers.

## Introduction

In natural selection theory, survival and reproduction are two fundamental tasks for all living creatures (Darwin, 1859). From the classic evolutionary perspective, offspring are a vehicle for their parents. When individuals survive to succeed in reproducing offspring, their genes can get transported to the next generation. However, modern evolutionary biologists posited that this classic fitness theory cannot fully explain the evolutionary mechanism, who further developed a series of new theories. These include inclusive fitness theory (Hamilton, 1964) and parental investment theory (Trivers, 1972). These modified theories articulate that humans act to preserve of their genes not merely through reproduction, but also through the act of helping and developing their offspring. Because of internal fertilization, females are 100 percent “sure” of their genetic contribution to offspring (Gaulin and Schlegel, 1980). In contrast, human males tend to invest less resources on offspring’s growth than do females, in that they face fierce competition in mating (Trivers, 1972), and that their mates may mate with other males (Burch and Gallup, 2000). In this way, males are faced with a risk that their putative children may not be their biological offspring (Buss, 2004; Larsen and Buss, 2008). Previous cross-cultural research suggested that men tended to invest more resources on offspring when perceiving a high certainty of paternity (Gaulin and Schlegel, 1980). As females are able to assure a blood relationship with their offspring, they are more likely to invest more resources in offspring than males (Trivers, 1972; Geary, 2000). From this point of view, maternal investment is unconditional, whereas paternal investment seems to be conditional (Trivers, 1972).

### Cues of Paternity

To cope with the uncertainty of paternity, male ancestors developed a series of unique adaptive mechanisms. Males seek various cues that would suggest genetic resemblance between father and child. The cues can be “indirect” as well as “direct.” (Flinn, 1988; Apicella and Marlowe, 2004; Bressan et al., 2009; Alvergne et al., 2010). Apicella and Marlowe (2004) maintained that spouse’s fidelity was an indirect index for males to assess paternity. If a male perceives infidelity of their spouse, he would feel depressed and anxious about the possibility that his offspring had been raised for other males, and the money and effort invested in their offspring were all “in vain” (DeSteno and Salovey, 1996; Harris and Christenfeld, 1996; Shackelford et al., 2002; Buss and Haselton, 2005; Edlund and Sagarin, 2009).

In additional to indirect cues, males use direct cues as well, such as facial resemblance and body odor, to assess their paternity (Bressan et al., 2009; Alvergne et al., 2010). Among these direct cues, facial resemblance has been widely studied by scholars. Previous studies found that when males looked more like their offspring, they tended to invest more affective and material resources in offspring and were less likely to commit domestic violence (Burch and Gallup, 2000; Alexandre et al., 2011). Biological studies have shown that craniofacial characteristics are highly hieratic (Johannsdottir et al., 2005). Previous studies have demonstrated that father-child facial resemblance can significantly predict the amount of paternal investment in fostering offspring. This notion has been demonstrated through various research methods, including using third party rating (Alvergne et al., 2009), self-report (Burch and Gallup, 2000; Apicella and Marlowe, 2004), or morphological method (DeBruine, 2005; Krupp et al., 2008). Generally, as paternity confidence relies on fathers’ perceived facial resemblance with their child, self-report measure has a relatively higher ecological validity than other research methods. Also, as morphological technology could manipulate the facial resemblance, a causality could be established in experimental researches. Thus, morphological method has a higher internal validity. In comparison with other two methods, the third party rating has a moderate ecological validity and internal validity.

### Parent–Child Facial Resemblance and Trait Anxiety

Facial resemblance is an important cue for males to assess paternity. If a male perceives low facial similarity with offspring, he may start to doubt his biological relationship with his child. This uncertainty of paternity would cause some psychological pressure and anxiety, which is defined as “an emotional state that included feelings of apprehension, tension, nervousness, and worry accompanied by physiological arousal” (Spielberger, 1983). In most cases, fatherhood is a stable and lifelong relationship. Influenced by social desirability, males are not willing to acknowledge an uncertainty of paternity, but instead will “bury their anxiety deep in heart”. After a long time, this psychological stress and nervousness would, eventually, shape a trait anxiety, which is a tendency to be anxious (Macleod et al., 1986; Williams et al., 1996). This is not the case for females. Since females can ensure a biological connection with their offspring (Gaulin and Schlegel, 1980), low mother–child facial resemblance does not make mothers feel stressful and nervous, which may not have an impact on trait anxiety. In sum, this study hypothesizes that parents’ gender moderates the relationship between perceived parent–child facial resemblance and parents’ trait anxiety.

### The Purpose of This Study

To foster ecological validity, this study recruited kindergarten students’ parents as participants, using a self-report method to explore the effect of perceived parent–child facial resemblance on trait anxiety. Considering that it is difficult to conduct a precise and reasonable composite score of parent–child facial resemblance in non-single child families, only parents from single child families were selected as the participants in this study. Furthermore, in order to avoid interference between father and mother, we only surveyed one parent of each family (father or mother).

Previous studies showed that the level of trait anxiety was influenced by age (Nakazato and Shimonaka, 1989), health condition, and social economic status (Kraus et al., 2011). In addition, in the Chinese cultural context, from a traditional view, male offspring, instead of females, are supposed to carry on the family line and take a responsibility of serving their aging parents while living and providing them with a proper burial after death (Peng, 2011), which makes many Chinese prefer sons over daughters (Lin and Zhao, 2014). Thus, in this study, the child’s gender may also influence parents’ trait anxiety. Taken together, in order to eliminate the effects of the variables stated above, this study considered these variables as control variables.

## Materials and Methods

### Participants

A total of 196 parents were initially recruited from a kindergarten in Beijing. In order to eliminate the possible effect of confounding variables, 151 parents (55 fathers and 96 mothers) from 151 independent single child families were selected as participants after removing 45 participants(31 from non-single child families, 3 from single parent families, and 13 participants did not respond). All of the participants were in their first marriage. Eight participants were minorities. The average age of participants was 34.83 years old (*SD* = 3.27). The participants’ average years of marriage was 8.49 years (*SD* = 4.26). The average age of the children was 4.66 years old (*SD* = 1.41), including 75 boys and 76 girls.

### Measures

#### Demographic Measure

Participants’ demographic information was gathered, including gender, age, years of marriage, the health status of participants and their spouses, and the educational attainment of participants and their spouses. Participants also reported the gender and health status of their youngest child, the number of children they had, and their annual household income.

#### Perceived Parent–Child Facial Resemblance Measure

A self-made perceived parent–child facial resemblance scale was used to measure participants’ facial resemblance between the parent and child. There were two items in this scale: “*In your opinion, how much does the youngest child look like you*?” and “*In your friends’ or family members’ views, how much does the youngest child look like you*?” The scale was rated on a 10-point Likert scale, ranging from 1 (*not at all*) to 10 (*completely*). The average score of two items was deemed as the index of parent-child facial resemblance. In this study, the correlation coefficient between these two items was 0.85.

#### Trait Anxiety Measure

The State-Trait Anxiety Inventory (STAI) (Spielberger, 1983) was used to measure participants’ trait anxiety. The STAI contains a total of 40 items rated on a 4-point Likert scale, ranging from 1 (*Not at all*) to 4 (*Very much*). The first twenty items measure respondents’ state anxiety, which are completed with regard to how the respondent felt at that very moment. The examples of items include “*I feel afraid*” and “*I am satisfied*”. The last twenty items measure respondents’ trait anxiety, which are completed with regard to how a respondent usually feels. Examples of items include “*I feel safe*”, “*I have lack of confidence*”, and “*I am a calm person*”. The last of the STAI was used to measure participants’ trait anxiety. The STAI has been demonstrated to have satisfactory reliability and validity: The test-retest reliability was.90 (Spielberger, 1983). The Cronbach’s α coefficient was 0.72 and the split-half reliability was 0.72 in this study.

### Procedures

This study was approved by Beijing Normal University Ethics Committee.

Research assistants delivered the questionnaires to kindergarten students in classrooms and instructed them to take the questionnaire to their mother or father. Each parent completed the questionnaires independently. At first, participants were required to fill out the demographic information questionnaire. Then, the participants completed the self-made parent–child facial resemblance scale to report their facial similarity with their child and the STAI to report the trait anxiety. Finally, after parents completed the questionnaire, the kindergarten students brought the completed questionnaires back to school and submitted them to the research assistants.

### Data Analyses

First, an independent sample *t*-test was conducted to examine the difference between father–child and mother–child facial resemblance. Second, a hierarchical regression analysis was conducted to investigate whether the effect of parent–child facial resemblance on parents’ trait anxiety differed based on parents’ gender. Finally, a simple slope effect test was conducted to further analyze how parents’ gender moderated the effect of perceived parent–child facial resemblance on trait anxiety.

### Ethics Statement

There are three reasons that we did not take ethics review. First, this study is only a simple survey research, in which we measured parents’ trait anxiety, parent-child facial resemblance, and demographic variables (e.g., gender, age). Our sample is the parents of kindergarten students, who are normal and healthy individuals. Therefore, this study did not involve vulnerable subjects or issues. Second, researchers delivered the questionnaires to kindergarten students in classrooms and instructed them to bring the questionnaires to their mothers or fathers. Parents completed the questionnaires independently and anonymously. Thus, they had right to decide whether they wanted to participate in this study. Finally, in the instruction, we emphasized that the data was only used for this study, and we would keep personal information confidential. As the reasons above have meet the requirements of ethics in psychological study, we believe that this study does not need to take ethics review beforehand. After the study was done, we submitted our research to Beijing Normal University ethics committee for review and the committee has approved this study.

## Result

### Preliminary Analyses

According to suggestions by Kraus et al. (2009), the average of standardized family income and the years of maternal and paternal education was used as a composite measure of participants’ social economic status (SES). To facilitate the subsequent regression analysis, gender of parents and children was coded (assigning “female” as “1” and “male” as “-1”) and the variables of parent–child facial resemblance and gender of parents were standardized.

### The Difference between Father– and Mother–Child Facial Resemblances

A *t*-test was conducted to analyze father– and mother–child facial resemblance, with the mid-point of 5.5 as the reference point. The results showed that both scores of father– and mother–child facial resemblance were significantly higher than the reference point, with a higher effect size for fathers than for mothers (father: *M* = 8.02, *SD* = 1.65; *t*_(53)_ = 11.21, *p* < 0.001, and Cohen’s *d* = 1.54; mother: *M* = 6.42, *SD* = 2.04; *t*_(95)_ = 4.41, *p* < 0.001, and Cohen’s *d* = 0.45). We conducted an independent sample *t*-test to analyze the differences between father– and mother–child facial resemblances. The result showed that the score of father–child facial resemblance was significantly higher than the score of mother-child facial resemblance (*t*_(148)_ = 4.98, *p* < 0.001, and Cohen’s *d* = 0.86).

### The Moderating Effect of Parent Gender on the Relationship between Parent–Child Facial Resemblance and Parents’ Trait Anxiety

We conducted a hierarchical regression analysis to explore the moderating effect of parent gender on the relationship between parent–child facial resemblance and parents’ trait anxiety. First, the control variables, including age, health status, spouse’s health status, child’s health Status, and family SES, were included into the regression equation. Second, parent gender and parent–child facial resemblance were included into the regression equation. Finally, the interaction between standardized parent–child facial resemblance and parents’ gender was included into the regression equation. As shown in **Table 1**, except for SES, the rest of the demographic variables (i.e., age, health status, spouse’s health state, and child’s health status) did not significantly predict parents’ trait anxiety. Additionally, parent–child facial resemblance significantly predicted trait anxiety. However, parent gender was not a significant predictor. The interaction between parent–child facial resemblance and parent gender significantly predicted parent trait anxiety.

**Table 1:** The effect of parent–child perceived facial resemblance (PFR) on parents’ trait anxiety.

	Predictor variable	Outcome variable: trait anxiety					
		
		Unstandardized regression coefficients					
		
		(First step)		(Second step)		(Third step)	
				
		*B*	*SE × B*	*B*	*SE × B*	*B*	*SE × B*
	Constant	1.654	–	1.825	–	1.989	–
First step	Age	<0.001	0.007	0.003	0.007	<0.001	0.007
	Health Status	0.035	0.058	0.044	0.063	0.060	0.062
	Health Status of Spouse	0.033	0.055	0.012	0.056	0.026	0.055
	Child’s Gender	-0.024	0.020	-0.027	0.020	-0.028	0.020
	Child’s Age	-0.016	0.015	-0.026	0.016	-0.024	0.015
	Health Status of Child	0.027	0.053	0.022	0.052	0.016	0.051
	Family SES	- 0.027^∗∗^	0.010	-0.030^∗∗^	0.010	-0.028^∗∗^	0.010
	*R^2^*	0.074					
Second step	Parent-Child PFR			- 0.027^∗∗∗^	0.011	-0.039^∗∗∗^	0.012
	Gender			-0.008	0.024	-0.027	0.025
	*R^2^*			0.117^∗^			
	*ΔR^2^*			0.043^∗^			
Third step	Parent-child PFR × Gender					0.028^∗^	0.012
	Adjusted *R^2^*					0.153^∗^	
	*ΔR^2^*					0.035^∗^	


*^∗∗∗^*p* < 0.001,^∗∗^*p* < 0.01, and ^∗^*p* < 0.05.*

**FIGURE 1:**
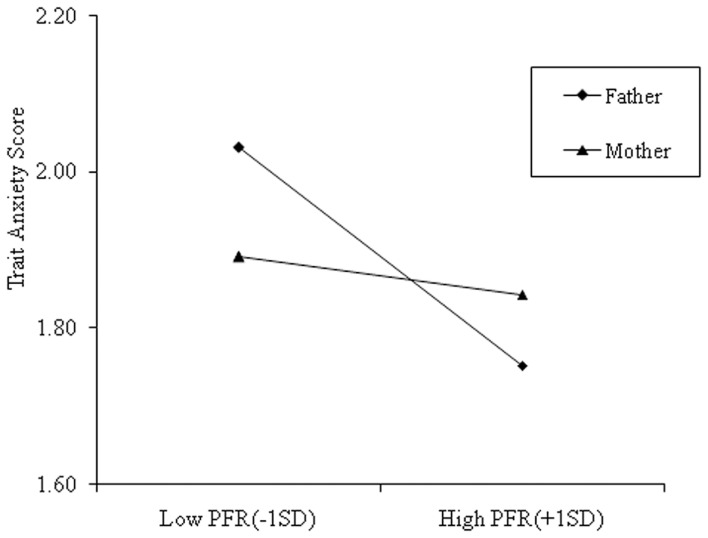
**The Moderating effect of parents’ gender on the relationship between perceived facial resemblance (PFR) and parents’ anxiety trait.** The figure depicts the trait anxiety score corresponding to -1SD and +1SD of PFR for both sexes. The slope was significant for men, but not for women.

As per the recommendations of Aiken et al. (1991), a simple slope effect test was conducted to further investigate the effect of the interaction between parent gender and parent–child facial resemblance on parents’ trait anxiety. As shown in **Figure 1**, for fathers, the effect of father–child facial resemblance on fathers’ trait anxiety was statistically significant (*B* = -0.068, *SE* = 0.020, *t*_(140)_ = -3.37, *p* = 0.001, and η^2^ = 0.08). For mothers, the effect of mother-child facial resemblance on mothers’ trait anxiety was not statistically significant (*B* = -0.012, *SE* = 0.013, *t*_(140)_ = -0.90, *p* = 0.36, and η^2^ < 0.01).

## Discussion

This study found that parent gender moderated the relationship between perceived parent–child facial resemblance and parents’ trait anxiety, which supported our hypotheses.

### Psychological Defense of Paternity Uncertainty

With regard to parents’ ratings of facial resemblance with their child, both rating scores of fathers and mothers were above the reference point, and males scored higher than females. These findings are inconsistent with the findings of previous studies (McLain et al., 2000; Bressan and Dal Martello, 2002). Employing a third party rating method, researchers did not find a significant difference between father– and mother–child facial resemblance (Bressan and Dal Martello, 2002), with one study even finding a significantly higher score for mothers relative to fathers (McLain et al., 2000). Conversely, in the present study, males reported that their children looked more like themselves than females. This might be because both males and females tend to display a self-serving bias—a belief that they are better than the average—which had been extensively demonstrated in various domains of social psychology (Lee and Waite, 2005; Kanten and Teigen, 2008; Krizan and Suls, 2008). Compared to females, males may have stronger self-serving biases in perceiving facial resemblance with their children. This may result from a psychological defense mechanism, which is used to cope with or reduce anxiety when fathers perceive uncertainty of paternity. Also, there is an alternative explanation: Women and their relatives may stress the resemblance between child and father in daily life, so as to obtain more resources from fathers (Regalski and Gaulin, 1993; McLain et al., 2000; Alvergne et al., 2007; Alvergne et al., 2010), and fathers pick up on those opinions (Burch and Gallup, 2000).

### Parent–Child Facial Resemblance and Trait Anxiety

People usually consider facial resemblance as a cue to ascertain a biological connection between individuals (Maloney and Dal Martello, 2006; Lewis, 2011). Because of its accessibility and convenience, fathers’ perceived facial resemblance with their child has a pivotal influence on their psychological experiences and behaviors. Previous studies have indicated that the degree of father-child facial resemblance has an impact on the amount of material and psychological resources that fathers invest in their offspring. The more similar the facial characteristics between a father and offspring, the more paternal resources he would invest (Alvergne et al., 2009), and the lower domestic violence rate would be (Burch and Gallup, 2000). Since young offspring are unable to support themselves, they depend on the custody and companionship provided by parents. To parents, facial resemblance with their child is a long-lasting stimulation. When perceiving a low facial resemblance with the children, a father will experience psychological pressure and nervousness, which, after a long time, may gradually turn into trait anxiety. In contrast, females do not have such an adaptive problem as do males. That may lead to the non-significant effect of mother-child facial resemblance on mother’s trait anxiety in this study. The present findings are consistent with the studies on sexual jealousy—specifically the notion that males felt more distressed and upset than females when imagining a sexual betrayal of their mate, which undermines paternity (Buss et al., 1992). Males will extricate themselves from the obsession with sexual betrayal by breaking up or divorcing with mates. Therefore, jealousy, as a response to sexual infidelity, is situational and transient. However, when males perceive their children not to be like them, they would result in doubts as to paternity. This uncertainty of paternity would lead to distressed and anxious feelings, which might turn into trait anxiety that after a long time of exposure.

Consistent with the results of Burch and Gallup (2000), the scores of item “In your opinion, how much does the youngest child look like you?” were highly correlated with the scores of “In your friends or family members’ views, how much does the youngest child look like you?”. (This study: *r* = 0.85, *n* = 170, and *p* < 0.001; Burch and Gallup (2000) study: *r* = 0.87, *n* = 81, and *p* < 0.001). Burch and Gallup (2000) noted that males’ evaluation of the phenotypic resemblance relied on “social mirror”, which reflected the opinions of their kin and friends (Daly and Wilson, 1982; Regalski and Gaulin, 1993). It suggests that males perceived confidence of paternity may not the actual paternity.

### Limitations and Future Research

Despite that the findings support the hypothesis that parent’s gender moderates the relationship between perceived parent–child facial resemblance and parents’ trait anxiety, there are several limitations in this study. First, this study uses a static correlational research paradigm, which could not establish a causal relationship. Therefore, future studies should improve internal validity from the following aspects: Priming methods or morphological technology can be adopted to manipulate the degree of parent–child facial resemblance to further examine the effect of parent–child facial resemblance on parents’ state anxiety. Second, considering that it is difficult to conduct a precise and reasonable composite score of parent–child facial resemblance in non-single child families, this study only adopts data from single child families. Further research needs to examine if the findings could extend to a different parent group with more than one child.

### Coda

The present findings suggest that perceived father–child facial resemblance, a direct cue for males to assess paternity, shapes fathers’ trait anxiety. As ascertain the maternity, this is not the case for females. Also, this study provides a new perspective to explore the development of trait anxiety, especially for adult males with offspring.

## Author Contributions

QY, QZ, JC, SJ, YQ, and WC designed this study and draft the manuscript; QY, YQ, and WC performed research; QY, QZ, JC, and SJ analyzed data. All atuthors approved the final version of manuscript for submission.

## Conflict of Interest Statement

The authors declare that the research was conducted in the absence of any commercial or financial relationships that could be construed as a potential conflict of interest.

The reviewer SW and handling Editor declared their shared affiliation, and the handling Editor states that the process nevertheless met the standards of a fair and objective review.
